# Symbiont Interactions in a Tripartite Mutualism: Exploring the Presence and Impact of Antagonism between Two Fungus-Growing Ant Mutualists

**DOI:** 10.1371/journal.pone.0008748

**Published:** 2010-01-15

**Authors:** Michael Poulsen, Cameron R. Currie

**Affiliations:** Department of Bacteriology, University of Wisconsin-Madison, Madison, Wisconsin, United States of America; Field Museum of Natural History, United States of America

## Abstract

Mutualistic associations are shaped by the interplay of cooperation and conflict among the partners involved, and it is becoming increasingly clear that within many mutualisms multiple partners simultaneously engage in beneficial interactions. Consequently, a more complete understanding of the dynamics within multipartite mutualism communities is essential for understanding the origin, specificity, and stability of mutualisms. Fungus-growing ants cultivate fungi for food and maintain antibiotic-producing *Pseudonocardia* actinobacteria on their cuticle that help defend the cultivar fungus from specialized parasites. Within both ant-fungus and ant-bacterium mutualisms, mixing of genetically distinct strains can lead to antagonistic interactions (i.e., competitive conflict), which may prevent the ants from rearing multiple strains of either of the mutualistic symbionts within individual colonies. The success of different ant-cultivar-bacterium combinations could ultimately be governed by antagonistic interactions between the two mutualists, either as inhibition of the cultivar by *Pseudonocardia* or *vice versa*. Here we explore cultivar-*Pseudonocardia* antagonism by evaluating *in vitro* interactions between strains of the two mutualists, and find frequent antagonistic interactions both from cultivars towards *Pseudonocardia* and *vice versa*. To test whether such *in vitro* antagonistic interactions affect ant colonies *in vivo*, we performed sub-colony experiments using species of *Acromyrmex* leaf-cutting ants. We created novel ant-fungus-bacterium pairings in which there was antagonism from one, both, or neither of the ants' microbial mutualists, and evaluated the effect of directional antagonism on cultivar biomass and *Pseudonocardia* abundance on the cuticle of workers within sub-colonies. Despite the presence of frequent *in vitro* growth suppression between cultivars and *Pseudonocardia*, antagonism from *Pseudonocardia* towards the cultivar did not reduce sub-colony fungus garden biomass, nor did cultivar antagonism towards *Pseudonocardia* reduce bacteria abundance on the cuticle of sub-colony workers. Our findings suggest that inter-mutualist antagonism does not limit what combinations of cultivar and *Pseudonocardia* strains *Acromyrmex* fungus-growing ants can maintain within nests.

## Introduction

Mutualistic symbioses are important drivers of host and symbiont evolution, specialization, and diversification, and have played key roles in shaping the evolution of life on Earth (e.g., [1]–[3]). Some of the key questions in understanding the ecology and evolution of mutualisms are what factors maintain the specificity and stability of mutualist pairings (e.g., [4]–[7]). Antagonistic interactions (conflict) occurring between different combinations of host and mutualist genotypes, or between genetically different strains of the same mutualist species, may affect and shape the specificity in host-symbiont pairings [8]–[14]. Recently, studies have illustrated that the ecological and evolutionary dynamics of bipartite mutualistic associations are shaped, at least in part, by the interactions they have within their broader ecological community (e.g., [6], [15]–[19]). This includes two studies that have illustrated that negative interactants have the potential to influence the stability of bipartite mutualisms [18]–[19]. Despite the recognition that bipartite mutualisms are influenced by antagonism and community dynamics, and often associate with additional mutualist lineages (e.g., [15], [20]–[23], the potential for within-host conflict between different beneficial symbionts affecting the stability and specificity of mutualist pairings has not been examined.

Fungus-growing ants engage in at least two mutualisms and have become a model system for studying the ecology and evolution of beneficial symbioses (e.g., [24]–[25]). These ants belong to the tribe Attini (Hymenoptera, Formicidae), a monophyletic group of more than 210 species that cultivate fungi for food [26]–[29]. Ant fungiculture is thought to have originated approximately 50 million years ago [30], and has evolved into an association where both ants and fungi are mutually interdependent (e.g. [31]): the fungus serves as the main food source for the ants, who in return provide a suitable growth environment for the fungus [32]–[34]. The cultivated fungi host microfungal parasites in the genus *Escovopsis*, which directly target and consume the mutualistic fungus and have the potential to be virulent [20; 35]. In addition to behavioral defenses that facilitate the physical removal of *Escovopsis* from ant gardens [36], fungus-growing ants maintain antibiotic-producing bacteria in the genus *Pseudonocardia* that produce secondary metabolites with antimicrobial activities against *Escovopsis* [34; 37–39]. *Pseudonocardia* is maintained on worker and queen cuticles, apparently supplied with nutrients through epicuticular bicellular gland secretions [40]. Other Actinobacteria have been found in queen pellets and fungus gardens of attine ants [41], as well as from worker cuticles [42], and some of these bacteria produce compounds capable of inhibiting *Escovopsis in vitro* [43]. However, more work is needed to establish their persistency and ecological role (cf. [25], [39]).

Both the fungal and bacterial mutualists are by default transmitted vertically (i.e., from parent to offspring colony): when a queen leaves for her mating flight, she carries a pellet of fungus, collected from her natal nest, in her infrabuccal pocket, and transports the mutualistic bacteria on the cuticle [34], [44]–[45]. Vertical uniparental transmission of mutualists from parent to daughter nests leads to the expectation of anciently propagated symbiont lineages that evolve in parallel with their lineages of ant hosts (cf. [9]–[10], [28]). Indeed, both microbial symbionts show some degree of broad-scale phylogenetic congruence with the ant host [26; 46–47]. Strict ant-symbiont phylogenetic congruence is, however, disrupted by symbiont switches (horizontal transmission) between fungus-growing ant colonies, species, and even genera [7], [48]–[53].

Horizontal transmission of microbial symbionts has the potential to result in mixing and competition between genetically different cultivar and *Pseudonocardia* strains. In *Acromyrmex*, competitive conflict between fungal strains is apparent, and the fungal cultivar defends its monopoly by imprinting ant fecal droplets with incompatibility compounds that aid in the detection of non-native fungal strains [14]. The ants facilitate this single-strain rearing through behavioral incompatibilities involving the removal of non-native fungal strains [49], [54]. Similarly, *Acromyrmex* ants appear to recognize and preferentially choose their resident *Pseudonocardia* over non-native strains [55]. This may facilitate the potential removal of non-native *Pseudonocardia* bacterial strains to prevent within-colony competition between strains, which has been shown to be present in *in vitro* Petri plate bioassay experiments [56]. Competition avoidance by the ants may explain why, at any given point in time, nests of *Acromyrmex* ants apparently maintain low strain diversity of *Pseudonocardia*: findings reported in [51] indicate that colonies of *A. octospinosus and A. echinatior* associate with a single strain (based on Elongation Factor Tu), while [42] found one *Acromyrmex octospinosus* colony that harbored two genetically different strains (based on 16S rDNA).

**Figure 1:**
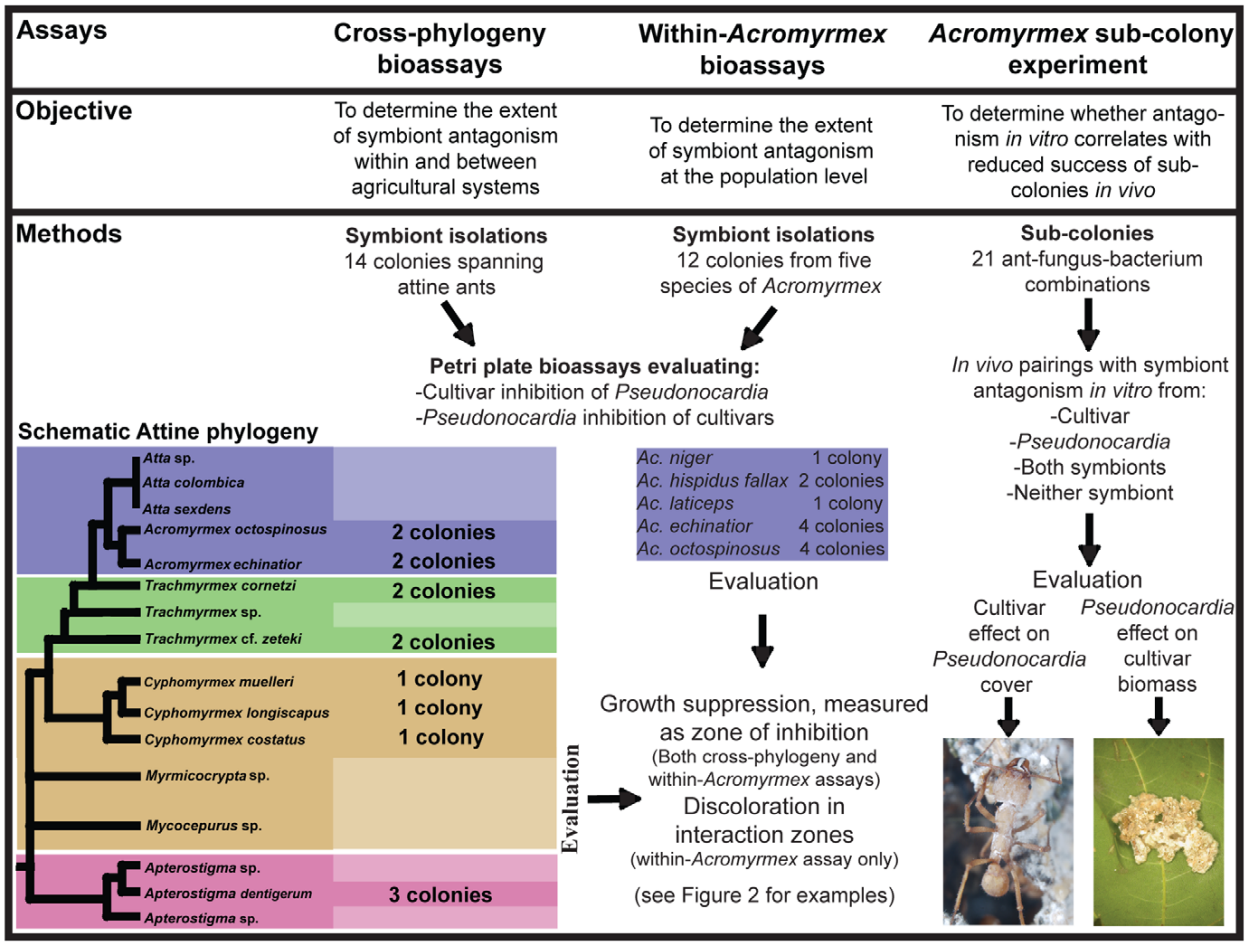
Schematic overview of the three components of our study: the cross-phylogeny bioassay experiments (left panel), the within-*Acromyrmex* bioassay experiments (middle panel), and the sub-colony evaluation of the role of *in vitro* antagonism on stability of novel *in vivo* sub-colony ant-fungus-bacterium combinations. For the cross-phylogeny bioassay experiments, the number of ant colonies for which cultivars and *Pseudonocardia* strains were obtained is indicated next to a schematic tree showing four of the five distinct attine agricultural systems: Paleoattine agriculture (pink), Neoattine lower agriculture (brown), Neoattine higher agriculture (green and purple), and leaf-cutter agriculture (purple) (see [30] for details). For the within-*Acromyrmex* bioassay experiments, the number of colonies from each of the five *Acromyrmex* species is likewise indicated. The right panel (sub-colony experiment) indicates the approach of the experiment: novel ant-fungus-bacterium combinations were performed in which there was directional antagonism from one, both, or none of the mutualists. The role of antagonism on stability was assessed by evaluating fungus garden mass and abundance of *Pseudonocardia* on ant cuticles (see main text for details).

Successful horizontal transmission of either the cultivar or *Pseudonocardia* will result in a reshuffling of ant-fungus-bacterium combinations within a nest. If this leads to antagonism between *Pseudonocardia* and the fungal cultivar, it may incur instability. We assess this by evaluating the extent and degree of antagonistic interactions between the cultivar and *Pseudonocardia*, as well as examine whether antagonism affects stability of ant-cultivar-bacterium pairings. Although a number of other fungi, yeasts, and bacteria are known from ant colonies [41]–[42], [57]–[60], their functional role and interactions with attine ants are less well understood; thus, our study focuses on the cultivar and *Pseudonocardia*. We evaluate the extent and degree of antagonistic interactions between the mutualists in Petri dish bioassay experiments. We test a collection of mutualists isolated from 14 ant colonies spanning most of the phylogenetic diversity of the associations (hereafter referred to as cross-phylogeny bioassays) (Fig. 1). In addition, we use 12 colonies of *Acromyrmex* to investigate symbiont interactions at the population level (hereafter referred to as within-*Acromyrmex* bioassays) (Fig. 1). To determine if the antagonistic interactions observed *in vitro* correlate with reduced success of sub-colonies *in vivo*, we perform sub-colony experiments composed of novel ant-fungus-bacterium pairings in which there is antagonism from one, both, or none of the symbionts (Fig. 1). In this experiment, we evaluate the effect of directional antagonism from *Pseudonocardia* towards cultivars by measuring changes in cultivar mass. We also examine the effect of antagonism from cultivars towards *Pseudonocardia* by measuring changes in the abundance of *Pseudonocardia* on the ant cuticle. Fig. 1 gives a schematic outline of the experiments performed; a more detailed flow diagram of our experimental approach is included as Supporting Information (Fig. S1).

## Materials and Methods

### Ant Colonies

For the cross-phylogeny bioassay experiments, we used cultivar and *Pseudonocardia* isolates from colonies spanning the diversity of attine ants: three colonies of *Apterostigma dentigerum* (a Paleoattine agriculture ant genus), one *Cyphomyrmex costatus*, one *C. muelleri*, and one *C. longiscapus* (a Neoattine lower agriculture ant genus); two *Trachymyrmex zeteki* and two *T. cornetzi* (a Neoattine higher agriculture non-leaf-cutting attine ant genus); and two colonies from *Acromyrmex echinatior* and two *A. octospinosus* (a Neoattine higher agriculture leaf-cutting ant genus) (cf. [30]) (Fig. 1). For the within-*Acromyrmex* bioassay experiments, we used cultivar and *Pseudonocardia* symbionts isolated from 12 colonies of sympatric and allopatric species of the leaf-cutting ant genus *Acromyrmex*. These included eight colonies of Panamanian *A. echinatior* and *A. octospinosus* colonies collected from 2001–2005 and four colonies of three ant species collected in Argentina in 2003 (one *A. niger*, two *A. hispidus fallax*, and one *A. laticeps* colony) (Fig. 1). See Table 1 for ant species, colony codes, and geographic origins.

**Table 1:** Fungus-growing ant colonies from which *Pseudonocardia* and cultivar isolates were obtained.

Ant species origin	Colony ID	Geographic origin
**Cross-phylogeny Petri dish bioassay**		
*Apterostigma dentigerum* (1)	AL040114-11	Panama
*Apterostigma dentigerum* (2)	MTP050505-10	Panama
*Apterostigma dentigerum* (3)	AL050512-17	Panama
*Cyphomyrmex muelleri* (4)	AL050512-19	Panama
*Cyphomyrmex longiscapus* (5)	ST040117-7	Panama
*Cyphomyrmex costatus* (6)	CC031210-9	Panama
*Trachymyrmex cornetzi* (7)	AL041002-3	Panama
*Trachymyrmex cornetzi* (8)	AL041005-10	Panama
*Trachymyrmex zeteki* (9)	AL030107-17	Panama
*Trachymyrmex zeteki* (10)	AL050513-4	Panama
*Acromyrmex octospinosus* (11)	UGM020518-5	Panama
*Acromyrmex octospinosus* (12)	CC031210-22	Panama
*Acromyrmex echinatior* (13)	CC031212-1	Panama
*Acromyrmex echinatior* (14)	CC031209-2	Panama
**Within-** ***Acromyrmex*** ** Petri dish bioassay**		
*Acromyrmex niger* (A)	CC030327-2	Argentina
*Acromyrmex hispidus fallax* (B)	SP030327-1	Argentina
*Acromyrmex hispidus fallax* (C)	UGM030327-2	Argentina
*Acromyrmex laticeps* (D)	UGM030330-4	Argentina
*Acromyrmex echinatior* (E)	291	Panama
*Acromyrmex echinatior* (F)	292	Panama
*Acromyrmex echinatior* (G)	295	Panama
*Acromyrmex echinatior* (H)	CC031212-1	Panama
*Acromyrmex octospinosus* (I)	CC011010-4	Panama
*Acromyrmex octospinosus* (J)	CC031210-22	Panama
*Acromyrmex octospinosus* (K)	ST040116-1	Panama
*Acromyrmex octospinosus* (L)	UGM020518-5	Panama

Cross-phylogeny strains are labeled 1–14 and within-*Acromyrmex* strains are labeled A–L (See Table 2).

### Cultivar and *Pseudonocardia* Isolations

For all ant colonies, the fungal cultivar was isolated by applying garden substrate directly onto Potato Dextrose Agar (PDA) (39 g/l; Becton, Dickinson & Co., MD 21151, USA); supplemented with 5 g/l of agar (Becton, Dickinson & Co., MD 21151, USA), added as a further solidifying agent, and Streptomycin sulphate and Penicillin-G (both at 12.5 mg/l), included to inhibit bacterial growth. Serial subculturing was performed to obtain pure cultures.

*Pseudonocardia* bacteria were isolated from *Apterostigma* and *Cyphomyrmex* ants either by grinding or by vortexing whole workers in 750 µl autoclaved water, and subsequently spreading 100–250 µl of this suspension onto chitin-agar plates containing antifungals [0.05 g Cycloheximide per liter of medium, and 0.04 g Nystatin dissolved in DMSO (2 g/l)]. For *Trachymyrmex* and *Acromyrmex*, bacteria were isolated from garden-tending workers carrying the largest abundance of *Pseudonocardia* on their cuticle [37; 61]. Bacteria were transferred to chitin-agar containing antifungals (concentrations as above), either by grinding or vortexing entire ants as described above, or by aseptically scraping the cuticle of the ants with a scalpel in areas where the bacterium was most abundant (the propleural plates). After 2–3 weeks of growth at room temperature, bacterial colonies were subcultured onto yeast malt extract agar (YMEA, 0.4% yeast extract, 1% malt extract, 0.4% dextrose) with antifungals (concentrations as above), and serial culturing was done until pure cultures were obtained. The bacterial strains used in this study represent the known phylogenetic diversity of *Pseudonocardia* associated with fungus-growing ants (for phylogenetic placement, see [55]–[56]).

### Petri Plate Bioassay Experiments

**Figure 2:**
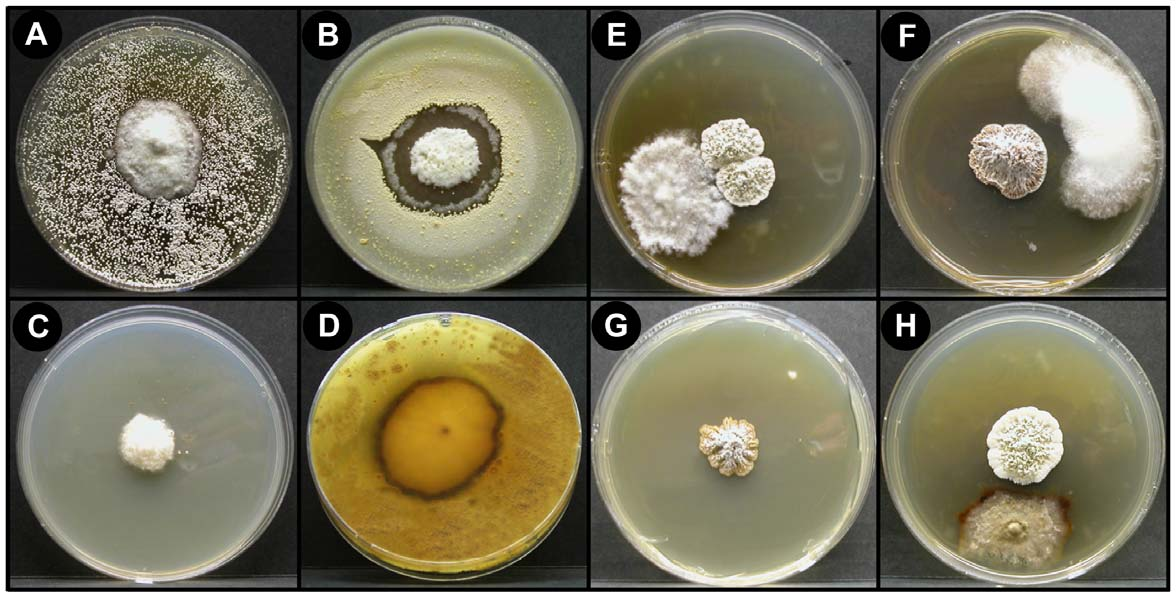
Micrographs showing typical extremes in reactions observed between symbiont pairings. (a)–(d) Cultivar (middle) inhibition of *Pseudonocardia* (edge) (a) No cultivar-induced inhibition of *Pseudonocardia*, (b) intermediary cultivar-induced inhibition of *Pseudonocardia*, (c) strong cultivar-induced inhibition of *Pseudonocardia*, (d) no cultivar-induced inhibition of *Pseudonocardia*, but strong discoloration indicating antagonistic chemical interactions. (e)–(h) *Pseudonocardia* (middle) inhibition of the cultivar (edge). (e) No *Pseudonocardia*-induced inhibition of the cultivar, (f) intermediary *Pseudonocardia*-induced inhibition of the cultivar, (g) strong *Pseudonocardia* induced inhibition of the cultivar, and (h) weak *Pseudonocardia*-induced inhibition of the cultivar, but strong discoloration indicating antagonistic chemical interactions.

Petri plate bioassay experiments testing for the presence of directional antagonism (cultivar inhibition of *Pseudonocardia* and *vice versa*) were performed for both cross-phylogeny and within-*Acromyrmex* symbiont isolates. See Fig. 2 for examples of observed interactions, including strong inhibition of one symbiont by another. All bioassay experiments were performed at a constant temperature of 25°C in 8.5 cm diameter Petri plates containing YMEA without antibiotics; three replicates were performed for all pairings.

#### Cultivar inhibition of actinobacterium

To evaluate the extent and degree of antagonism from the cultivar towards *Pseudonocardia*, the cultivar was inoculated in the middle of the Petri plate and allowed to grow for one month. *Pseudonocardia* was then inoculated to the entire unoccupied Petri dish area by applying 200 µl of autoclaved water containing a suspension of bacterial cells. One week after *Pseudonocardia* was applied the minimum zone of inhibition (ZOI) between the two was measured. For the cross-phylogeny assay we ran 588 bioassays (i.e., 14 cultivar strains tested against 14 *Pseudonocardia* strains replicated three times), while in the within-*Acromyrmex* bioassay 432 assays were conducted (i.e., 12 cultivar strains tested against 12 *Pseudonocardia* strains replicated three times). In the within-*Acromyrmex* bioassay, medium discoloration, suggesting antagonistic chemical interactions between symbiont pairs (cf. [62]–[63]), was noted.

#### Actinobacterium inhibition of cultivar

To evaluate the possible inhibition of cultivar by *Pseudonocardia*, the actinobacterium was point inoculated in the middle of the Petri plate and allowed to grow for two weeks. The cultivar was then point inoculated at the edge of the plate according to previously established methods (e.g., [39], [64]). After two months the minimum ZOI was measured. As for the cultivar-*Pseudonocardia* assays described above, the cross-phylogeny bioassay included 14 *Pseudonocardia* strains tested against 14 cultivar strains (replicated three times), while the within-*Acromyrmex* bioassay involved 12 *Pseudonocardia* strains tested against 12 cultivar strains (replicated three times). Because of the high proportion of pairings in which there was cultivar inhibition in the cross-phylogeny bioassay (see Results; Table 2), we performed two separate within-*Acromyrmex* Petri plate bioassay experiments. The first bioassay used the same protocol as the cross-phylogeny bioassay, and resulted in comparable and very strong growth inhibition of the majority of cultivar strains (See Results). To evaluate whether inhibition was generally weaker if the *Pseudonocardia* mass was smaller, i.e., the bacterium had less time to secrete secondary metabolites before interacting with the cultivar, we performed a second bioassay in which we inoculated the cultivar one week prior to *Pseudonocardia* inoculation. This allowed the cultivar to grow enough for us to document more variation in the degree of inhibition of different cultivars by different *Pseudonocardia* strains, without having the cultivar growing to an extent that it would inhibit *Pseudonocardia*. As above, discoloration was noted when present.

**Table 2:** Combinations used in the sub-colony experiment evaluating whether the presence/absence of antagonism impacts the success of ant fungiculture.

		Presence of antagonism (Mean ZOI in cm; n = 3)			
Colony of origin of ants and *Pseudonocardia*	Colony-of-origin of cultivar	Degree of antagonism from cultivar	Degree of antagonism from *Pseudonocardia* (no head start)	Degree of antagonism from *Pseudonocardia* (with head start)	Direction of Conflict*
A	K	0	2.0	0	None
	D	0.20	1.4	0	Cultivar
	A (control)	0	2.0	0.40	Bacterium
C	J	0.10	2.2	0	Cultivar
	D	0.10	2.4	0	Cultivar
	C (control)	0	2.2	0	None
D	E	0	1.8	0	None
	J	0.10	2.6	0	Cultivar
	D (control)	0.10	2.0	0	Cultivar
C	I	0.10	2.2	0.60	Both
	D	0.10	2.4	0	Cultivar
	C (control)	0	2.2	0	None
H	I	0.10	1.6	0.63	Both
	K	0	1.4	0	None
	H (control)	0.30	1.9	0	Cultivar
A	I	0	2.1	0.60	Bacterium
	D	0.20	1.4	0	Cultivar
	A (control)	0	2.0	0.40	Bacterium
F	I	0.20	1.3	0	Cultivar
	D	0.33	1.7	0.50	Both
	F (control)	0.70	1.6	0.57	Both

*Based on the *Pseudonocardia*-cultivar inhibition bioassay where the cultivar was given a head start, since the vast majority of pairings in the former bioassay (no head start) displayed some inhibition.

The column on the far right indicates the direction of antagonism, from either cultivar towards *Pseudonocardia* (cultivar), from *Pseudonocardia* towards cultivar (bacterium), or from neither (none) or both (both) of the symbionts. See Table 1 for species and geographical origin of colonies.

### Sub-Colony Evaluation of the Effect of Symbiont Antagonism on Colony Success

To evaluate the potential effects of symbiont antagonism on the success of fungus-growing ant colonies, we tested a total of 21 ant-bacterium-fungus combinations using five of the colonies from which symbionts used in the within-*Acromyrmex* bioassay experiments originated. The combinations selected included ones in which we had observed either high or low levels of antagonism between pairings in the Petri plate bioassays (see Table 2 for colony combinations). Antagonism could thus be either high or low and be from either cultivars towards *Pseudonocardia*, from *Pseudonocardia* towards cultivars, from both symbionts, or from neither symbiont (Table 2). Three sub-colonies were set up for each combination and changes in fungus garden weight and *Pseudonocardia* abundance on the cuticle of major workers in sub-colonies was evaluated.

Sub-colonies were created using previously developed methods that have proven useful for evaluating the short-term success of ant-fungus combinations despite being substantially smaller than mature nests (e.g., containing 4–8 workers compared to mature nests with tens of thousands of workers: [14], [39], [49], [54]). Sub-colonies were set up in 60 ml plastic cups with small holes in the lids to provide air, moist tissue to assure high humidity, and an oak leaf to provide forage for the ants (cf. [14], [39], [49]). Each plastic cup contained 150±1 mg of fungus garden, three minor workers, and three major workers covered with *Pseudonocardia* bacteria (abundance scores 10–12 in [65]). All visible larvae and pupae were removed, but eggs and smaller larvae that could not readily be removed were left in the sub-colony fungus gardens, which aids in the ants continuing their normal tasks of foraging, fungus garden maintenance, and caring for the brood (cf. e.g., [48]). In order to overcome ant-fungus behavioral incompatibility, known to last for up to 10–14 days after ants are first provided with a fungus fragment genetically different from their original fungus [14], [49], we provided 150±1 mg of fresh fungus material from source colonies daily for the first ten days. After ten days, ants in all sub-colonies maintained their fungus gardens without any signs of behavioral ant-fungus incompatibilities. During fungus garden replacement, the wet weight of the old fungus fragment was determined and clean moist tissue and a fresh oak leaf were provided.

After ten days, we stopped providing fresh fungus material, and instead only weighed sub-colony fungus gardens to assess interaction stability between different ant-fungus-bacterium combinations. During each of these weighing sessions, we scored the abundance of *Pseudonocardia* on the cuticle of the major workers using a previously developed semi-quantitative scale going from 0 (no bacteria visible) to 12 (complete and dense cover of bacteria) on the cuticle of major workers of *Acromyrmex* [65]. We did not do this during the first ten days because of the potential confounding effects of ant-fungus behavioral incompatibilities, but rather did this daily from day 11 to day 21 and every second day from day 21 to day 33. We are aware that ant colony/genotype might have affected the abundances observed, but at the moment it is not possible to eliminate this effect, because a method for switching *Pseudonocardia* has yet to be developed. At day 33, all sub-colonies had crashed so the experiment was terminated.

The overall effects of the ant-fungus-bacterium combinations on fungus weight and *Pseudonocardia* abundance, averaged over the three major workers present per sub-colony, were tested using repeated measures ANOVA tests performed using JMP IN 5.1 [66]. Comparisons included the following main factors: i) colony-of-origin of the ant (nominal), colony-of-origin of the fungus (nominal); ii) time since start of the experiment (nominal); iii) degree of *Pseudonocardia* inhibition of the cultivar (continuous, in cm ZOI); and iv) degree of cultivar inhibition of *Pseudonocardia* (continuous, in cm ZOI). In addition, interaction terms between ant colony of origin and time, and fungus colony of origin and time were included based on stepwise examination. Two analyses were performed on the effects on fungus mass: one including the entire run of the experiment, i.e., both during the first ten days where we provided fresh fungus material daily and from day 11–33 where we stopped provisioning of fungus material; the second test included only the time period in which we did not provide fresh fungus material (days 11–33). Because actinobacterium abundance scores were only obtained from day 11 and onwards, and because of missing values after day 21 in some pairings (See Tables 3, 4), the repeated measures ANOVA was performed only on days 11–21.

**Table 3:** Results of three ANOVAs testing the effects of ant origin, fungus origin, time since start of the experiment, directional antagonism from cultivars and *Pseudonocardia*, respectively, as well as the interaction between ant colony-of-origin and fungus colony-of-origin by time, on fungus weight (left) and actinobacterium coverage of ants (right).

	Fungus garden mass						Actinobacterium abundance		
	Full duration of the experiment			From day 11 and onwards			From day 11–21		
Effect	F	df	p	F	df	p	F	df	p
Ant origin	2.728	4	0.0281	1.587	4	0.1759	159.9	4	<0.0001
Fungus origin	63.23	8	<0.0001	66.94	8	<0.0001	19.85	8	<0.0001
Time	424.1	26	<0.0001	379.4	15	<0.0001	60.22	10	<0.0001
Ant origin * Time	1.128	104	0.1870	1.192	60	0.1576	3.062	40	<0.0001
Fungus origin * Time	6.465	208	<0.0001	5.732	120	<0.0001	1.103	80	0.2648
Inhibition of *Pseudonocardia* by cultivar	0.0586	1	0.8088	0.0788	1	0.7789	0.8391	1	0.3601
Inhibition of cultivar by *Pseudonocardia*	0.2028	1	0.6526	0.0913	1	0.7626	10.70	1	0.0011

Because actinobacterium abundance scores were only obtained from day 11 and onwards, and because of missing values, we performed the ANOVA on days 11–21 only.

**Table 4:** Results of ANOVAs on each of the seven individual combinational experiments listed in Table 2.

	Fungus garden mass						Actinobacterium abundance		
	Full duration of the experiment			From day 11 and onwards			From day 11 and onwards		
Effect	F	df	p	F	Df	p	F	df	p
**Ants from colony A on fungus from colony A, K or D**									
Ant-fungus combination	122.5	2	<0.0001	96.01	2	<0.0001	109.4	2	<0.0001
Time	83.07	15	<0.0001	71.04	26	<0.0001	6.035	10	<0.0001
Ant-fungus combination*Time	9.310	30	<0.0001	8.883	52	<0.0001	1.869	20	0.0304
**Ants from colony C on fungus from colony C, J, or D**									
Ant-fungus combination	4.106	2	0.0195	4.403	2	0.0137	31.58	2	<0.0001
Time	59.23	15	<0.0001	70.57	26	<0.0001	15.95	10	<0.0001
Ant-fungus combination*Time	1.364	30	0.1309	1.371	52	0.0706	1.479	20	0.1201
**Ants from colony D on fungus from colony D, E, or J**									
Ant-fungus combination	5.002	2	0.0086	7.221	2	0.0010	8.274	2	0.0006
Time	45.85	15	<0.0001	57.37	26	<0.0001	7.483	10	<0.0001
Ant-fungus combination*Time	1.794	30	0.0174	1.793	52	0.0031	1.197	20	0.2866
**Ants from colony C on fungus from colony C, I, or D**									
Ant-fungus combination	4.151	2	0.0187	3.616	2	0.0291	11.02	2	<0.0001
Time	118.0	15	<0.0001	135.6	26	<0.0001	30.01	9	<0.0001
Ant-fungus combination*Time	1.079	30	0.3783	1.129	52	0.2802	0.946	18	0.5301
**Ants from colony H on fungus from colony H, I, or K**									
Ant-fungus combination	1.965	2	0.1457	3.992	2	0.0203	0.8979	2	0.4123
Time	235.2	15	<0.0001	247.7	26	<0.0001	10.38	10	<0.0001
Ant-fungus combination*Time	0.8174	30	0.7306	0.7756	52	0.8556	0.4239	20	0.9826
**Ants from colony A on fungus from colony A, I, or D**									
Ant-fungus combination	153.2	2	<0.0001	136.0	2	<0.0001	7.125	2	0.0017
Time	65.15	15	<0.0001	67.32	26	<0.0001	22.83	9	<0.0001
Ant-fungus combination*Time	9.602	30	<0.0001	11.28	52	<0.0001	1.470	18	0.1338
**Ants from colony F on fungus from colony F, I, or D**									
Ant-fungus combination	0.4526	2	0.6373	0.4943	2	0.6109	0.7743	2	0.4656
Time	233.0	15	<0.0001	239.8	26	<0.0001	22.17	9	<0.0001
Ant-fungus combination*Time	0.6268	30	0.9270	0.6330	52	0.9715	0.4457	18	0.9702

The main factors ant-fungus pairing (nominal) and time (nominal) are included, in addition to the interaction between ant-fungus pair and time. Due to missing data as sub-colonies started to crash, the ANOVAs evaluating actinobacterium abundances could not be done over the entire duration of the experiment. Thus, for pairings A on fungus from colony A, K or D, C on fungus from colony C, J, or D, D on fungus from colony D, E, or J, and H on fungus from colony H, I, or K only days 11–23 were included. For the remaining pairings (C on fungus from colony C, I, or D; A on fungus from colony A, I, or D; and F on fungus from colony F, I, or D), tests were performed only on data from days 11–21.

To determine if certain ant-fungus combinations involving ants from the same colony behaved differently than ants from other colonies, we performed repeated measures ANOVAs for each of the 7 experimental combinations performed (Table 2). This provided a more detailed test of the role of antagonism on fungus weight and *Pseudonocardia* abundance than the tests of overall effects of ant-fungus-bacterium pairings on fungus weight and actinobacterium abundance described above. The tests were also performed using JMP IN 5.1 [66], with factors including ant-fungus combination (nominal), time since start of the experiment (nominal), and the interaction between ant-fungus combination and time. As above, two analyses were performed to test the effect on fungus mass: one including the entire run of the experiment and one including days 11–33 only. Because actinobacterium abundance scores were only obtained from day 11 and onwards, and because of missing values, we performed the ANOVAs on days 11–21 for some combinations and days 11–23 for others (see Table 4 for details).

## Results

### Petri Plate Bioassay Experiments

#### Cultivar inhibition of actinobacterium

**Figure 3:**
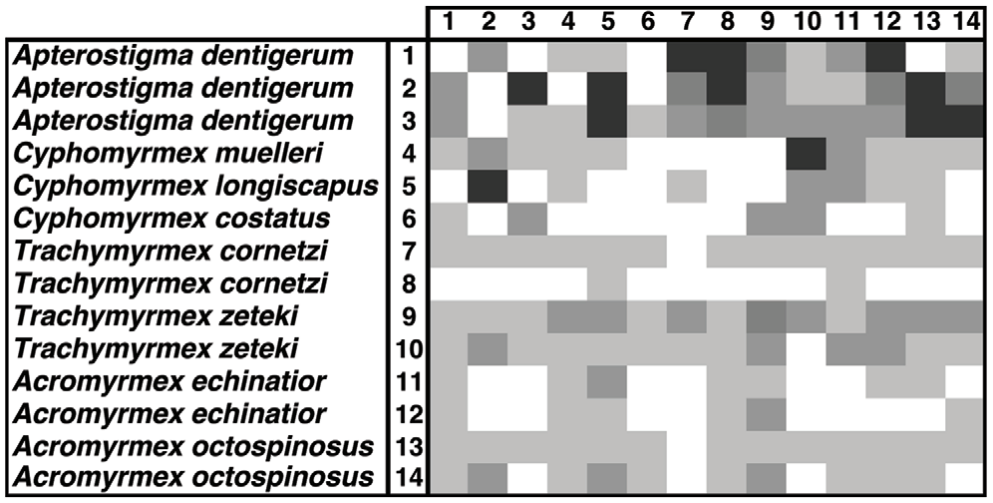
Diversity of interactions in bioassays examining the presence and degree of cultivar inhibition of *Pseudonocardia* for symbionts isolated from across the phylogenetic diversity of the ant-fungus-bacterium association. Each box represents the average zone of inhibition (ZOI; n = 3) of a given pairing and different shades of grey indicate the degree of inhibition: White: ZOI = 0 cm, Light grey: ZOI = 0.01−0.29 cm, Grey: ZOI = 0.30−0.59 cm, Darker grey: ZOI = 0.60−0.89 cm, and Darkest grey: ZOI>0.90 cm.

**Figure 4 pone-0008748-g004:**
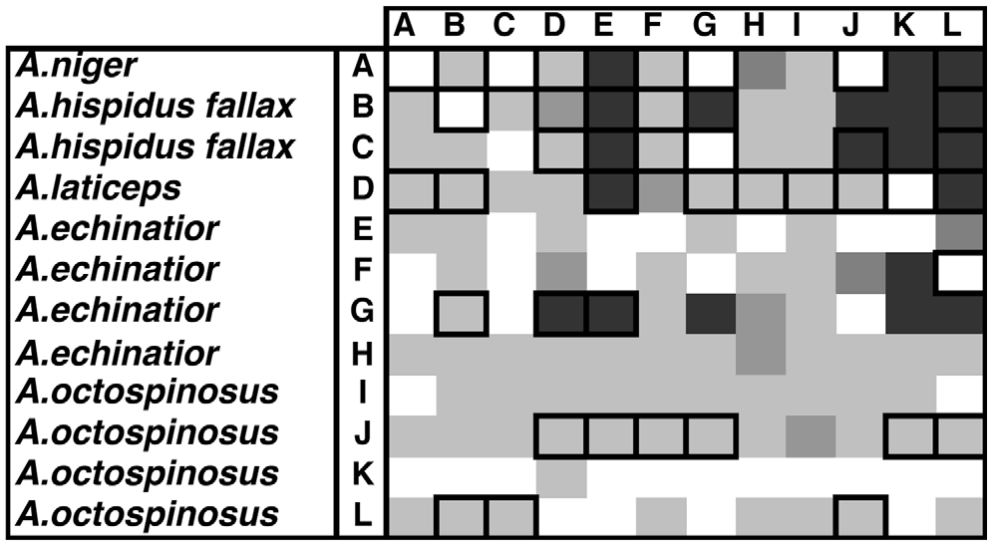
Diversity of interactions in bioassays examining the presence and degree of cultivar inhibition of *Pseudonocardia* between symbionts isolated from five species within the genus *Acromyrmex*. Each box represents the average zone of inhibition (ZOI; n = 3) of a given pairing and different shades of grey indicate the degree of inhibition: White: ZOI = 0 cm, Light grey: ZOI = 0.01−0.29 cm, Grey: ZOI = 0.30−0.59 cm, Darker grey: ZOI = 0.60−0.89 cm, and Darkest grey: ZOI>0.90 cm. Frames around boxes indicate pairings in which dark coloration suggesting antagonistic chemical interactions between symbionts were observed.

**Figure 5 pone-0008748-g005:**
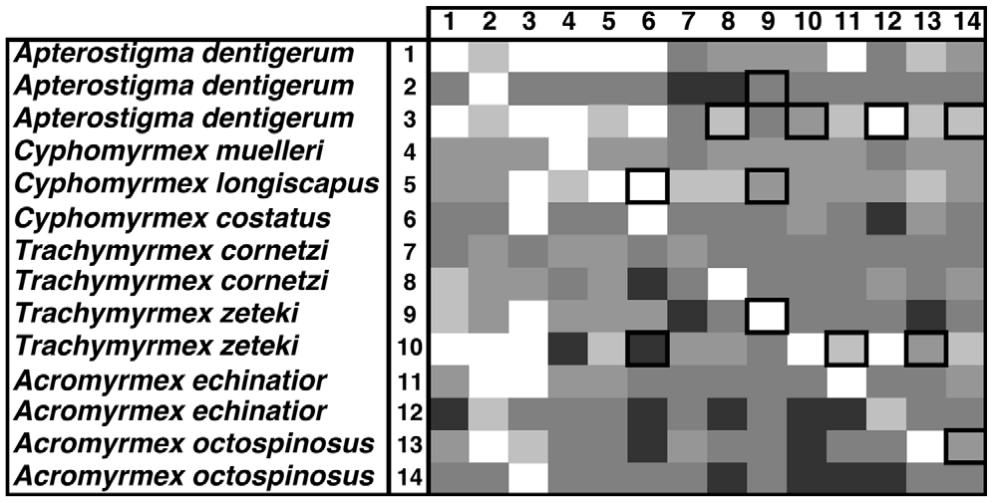
Diversity of interactions in bioassays examining the presence and degree of *Pseudonocardia* inhibition of cultivars for symbionts isolated from across the phylogenetic diversity of the ant-fungus-bacterium association. Each box represents the average zone of inhibition (ZOI; n = 3) of a given pairing and different shades of grey indicate the degree of inhibition: White: ZOI<0.50 cm, Light grey: ZOI = 0.51−1.00 cm, Grey: ZOI = 1.01−1.50 cm, Darker grey: ZOI = 1.51−2.00 cm, and Darkest grey: ZOI>2.01 cm.

Inhibition of *Pseudonocardia* by the fungal cultivar was observed in 68.4% and 72.9% of the bioassay pairings in our cross-phylogeny (Fig. 3) and within-*Acromyrmex* experiments (Fig. 4), respectively. The extent of inhibition of *Pseudonocardia* by cultivars ranged from 0 to 2.83 cm ZOI (Fig. 2A–D), but inhibition was generally low (mean±SE: 0.28±0.05 cm and 0.36±0.06 cm for the cross-phylogeny and within-*Acromyrmex* bioassays, respectively; Figs. 3 and 4). In the cross-phylogeny bioassay, individual strains of the cultivar inhibited on average 10 *Pseudonocardia* strains (range 2 to 14) and individual *Pseudonocardia* strains were on average susceptible to inhibition from 10 cultivar strains (range 6 to 12). Similarly, in the within-*Acromyrmex* bioassay, individual cultivars inhibited on average nine *Pseudonocardia* strains (range 1 to 12), while individual *Pseudonocardia* strains were susceptible to inhibition by an average of 9 cultivars (range 6 to 11). In the within-*Acromyrmex* bioassay, discoloration was observed in 31.3% of pairings. This occurred primarily in pairings with little growth inhibition, and mainly involved a few specific strains of cultivar, which frequently exhibited discoloration across several of the strains of *Pseudonocardia* (Fig. 5).

#### Actinobacterium inhibition of cultivar

**Figure 6:**
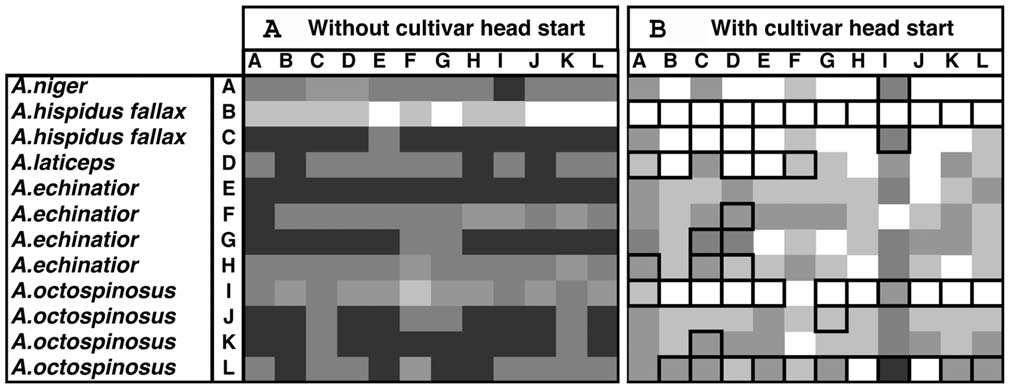
The results of two bioassays examining the presence and degree of *Pseudonocardia* inhibition of cultivars between symbionts isolated from five species within the genus *Acromyrmex*. Each box represents the average zone of inhibition (ZOI; n = 3) of a given pairing and different shades of grey indicate the degree of inhibition. (a) Shows the bioassay results for when *Pseudonocardia* was inoculated two weeks prior to the inoculation of the cultivar: White: ZOI<0.50 cm, Light grey: ZOI = 0.51−1.00 cm, Grey: ZOI = 1.01−1.50 cm, Darker grey: ZOI = 1.51−2.00 cm, and Darkest grey: ZOI>2.01 cm. (b) Shows the same bioassay pairings but in these pairings, the cultivar was inoculated one week prior to *Pseudonocardia*: White: ZOI = 0 cm, Light grey: ZOI = 0.01−0.29 cm, Grey: ZOI = 0.30−0.59 cm, Darker grey: ZOI = 0.60−0.89 cm, and Darkest grey: ZOI>0.90 cm. Frames around boxes indicate pairings in which dark coloration was observed suggesting antagonistic chemical interactions between symbionts.

Inhibition of the fungal cultivar by *Pseudonocardia* tended to be much stronger than inhibition of *Pseudonocardia* by the cultivar. In the cross-phylogeny bioassay, where *Pseudonocardia* was inoculated two weeks prior to the cultivar, there was some degree of inhibition in 91.8% of pairings (mean±SE ZOI: 1.34±0.05 cm) (Fig. 5). Discoloration was observed in very few pairings (5.6%), all of which had relatively small zones of inhibition. In the first within-*Acromyrmex* bioassay, using the same setup as in the cross-phylogeny bioassay, 96.5% of pairings displayed some inhibition (mean ZOI±SE: 1.90±0.04 cm) (Fig. 6A). When the cultivar was inoculated one week prior to *Pseudonocardia* inhibition was less frequent (66.0%; mean ZOI±SE: 0.20±0.02 cm) (Fig. 6B). As in the cross-phylogeny bioassay, the presence of discoloration (34.7% of pairings) was observed only in pairings with small zones of inhibition (i.e., when there was substantial growth of the cultivar) and appeared to be present primarily in pairings involving three of the *Pseudonocardia* strains (Table 1: B, I, or L).

### Evaluating the Effect of Symbiont Antagonism on Colony Success

#### Effect of *Pseudonocardia* antagonism on fungus garden weight

**Figure 7:**
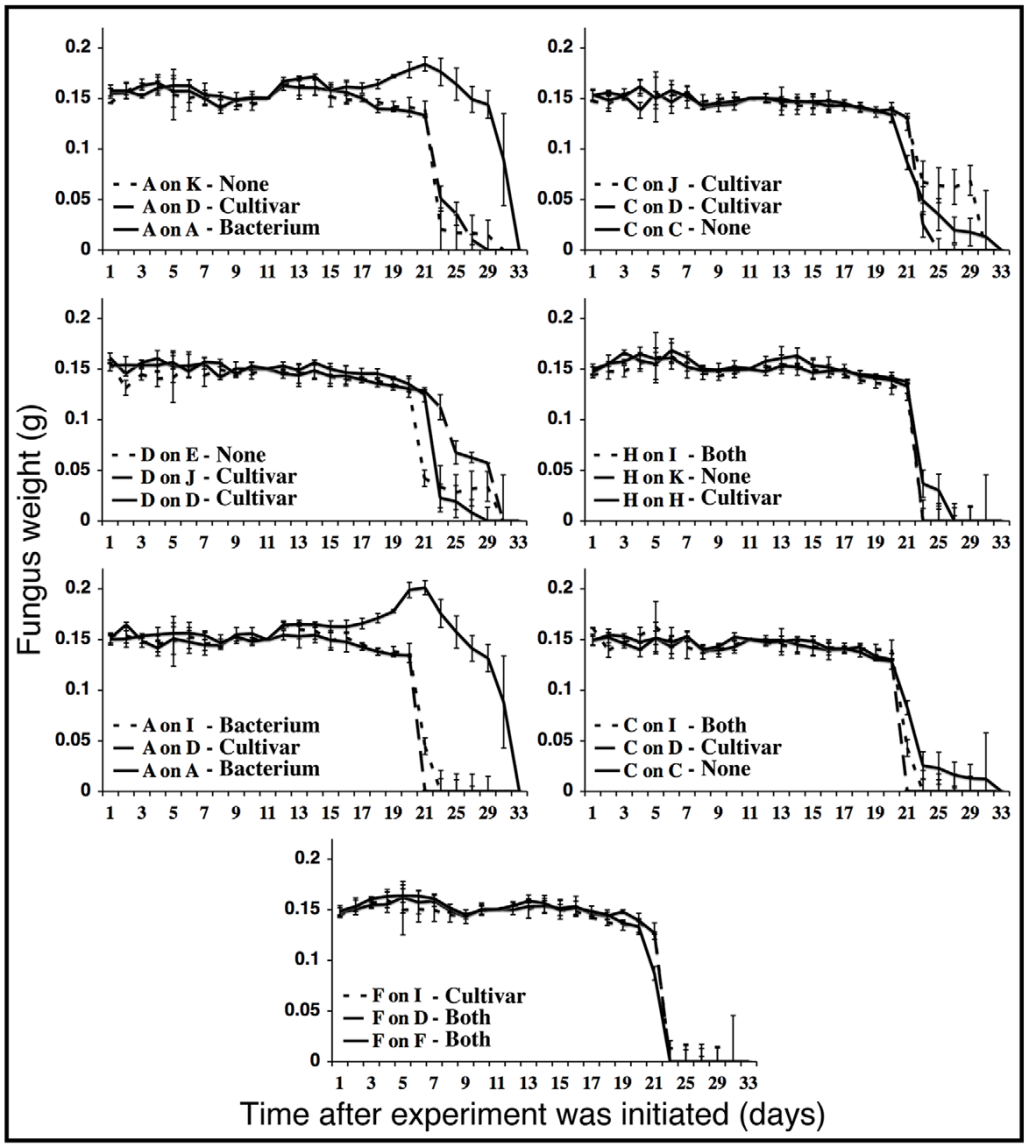
The results of the sub-colony experiment evaluating the effect of *Pseudonocardia* antagonism on sub-colony fungus garden weight. Means±SE of three sub-colonies are given. During the first 10 days of the experiment, sub-colonies were provided 150±1 mg of fresh fungus material daily in order to allow sub-colony workers to accustom to the new fungus garden material. From day 11–33, the fungus garden mass was weighed, but not replaced with new fungus material. The direction of antagonism observed *in vitro* is indicated next to the legend, either as None (antagonism from neither symbiont), Cultivar (antagonism from the cultivar towards *Pseudonocardia*), Bacterium (antagonism from *Pseudonocardia* towards the cultivar), or Both (antagonism from both symbionts).

All sub-colonies initiated a fungus garden with the fungus fragment provided, irrespective of the colony of origin, and remained relatively stable, i.e., without signs of strong ant-fungus behavioral incompatibilities (cf. [49]), during the first 10 days when they were provided fresh fungus material from source colonies daily (Fig. 7). All sub-colonies also remained stable from day 11 to 21, during which the fungus garden fragments were not replaced daily (Fig. 7). Thereafter fungus gardens started gradually declining, although the pace of this decline varied between ant-fungus-bacterium combinations (Fig. 7). Table 3 gives the results of two repeated measures ANOVAs testing the factors affecting fungus garden weight, both for the entire duration of the experiment and from day 11 and onwards only. These analyses found significant effects of both the colony origin of the fungus material (Day 1–33: F_8_ = 63.23, p<0.0001; Day 11–33: F_8_ = 66.94, p<0.0001), time (Day 1–33: F_26_ = 424.1, p<0.0001; Day 11–33: F_15_ = 379.4, p<0.0001), and the colony-of-origin and time interaction (Day 1–33: F_208_ = 6.465, p<0.0001; Day 11–33: F_120_ = 5.732, p<0.0001) (Table 3). The ant colony-of-origin involved significantly affected fungus weight when evaluated across the entire duration of the experiment (F_4_ = 2.728, p = 0.0281); however, this effect disappeared when evaluating only day 11–33 (F_4_ = 1.587, p = 0.1759). Time was the only statistically significant effect on fungus garden mass in all seven experimental combinations, and this effect was present irrespective of whether evaluated over the entire course of the experiment or only between day 11 and day 33 (Table 2, 4). Furthermore, the ant-fungus combination had significant effects on fungus garden mass in five of seven experiments when the entire duration of the experiment was considered and in six of the seven experiments when only days 11–33 were considered (Table 4; Fig. 7). There were also significant interaction effects between ant-fungus combination and time in some pairings (Table 4). There was no overall statistically significant effect of the degree of *Pseudonocardia* inhibition of the cultivar *in vitro* on fungus weight (F_1_ = 0.2028, p = 0.6526) (Fig. 7; Table 3). Rather, the ant-fungus-bacterium combination with the largest fungus garden mass at the end of the experiment was in fact a combination with *Pseudonocardia* inhibition of the cultivar *in vitro* (ants and fungi both from colony A) (Fig. 7).

#### Effect of cultivar antagonism on the abundance of *Pseudonocardia*

**Figure 8:**
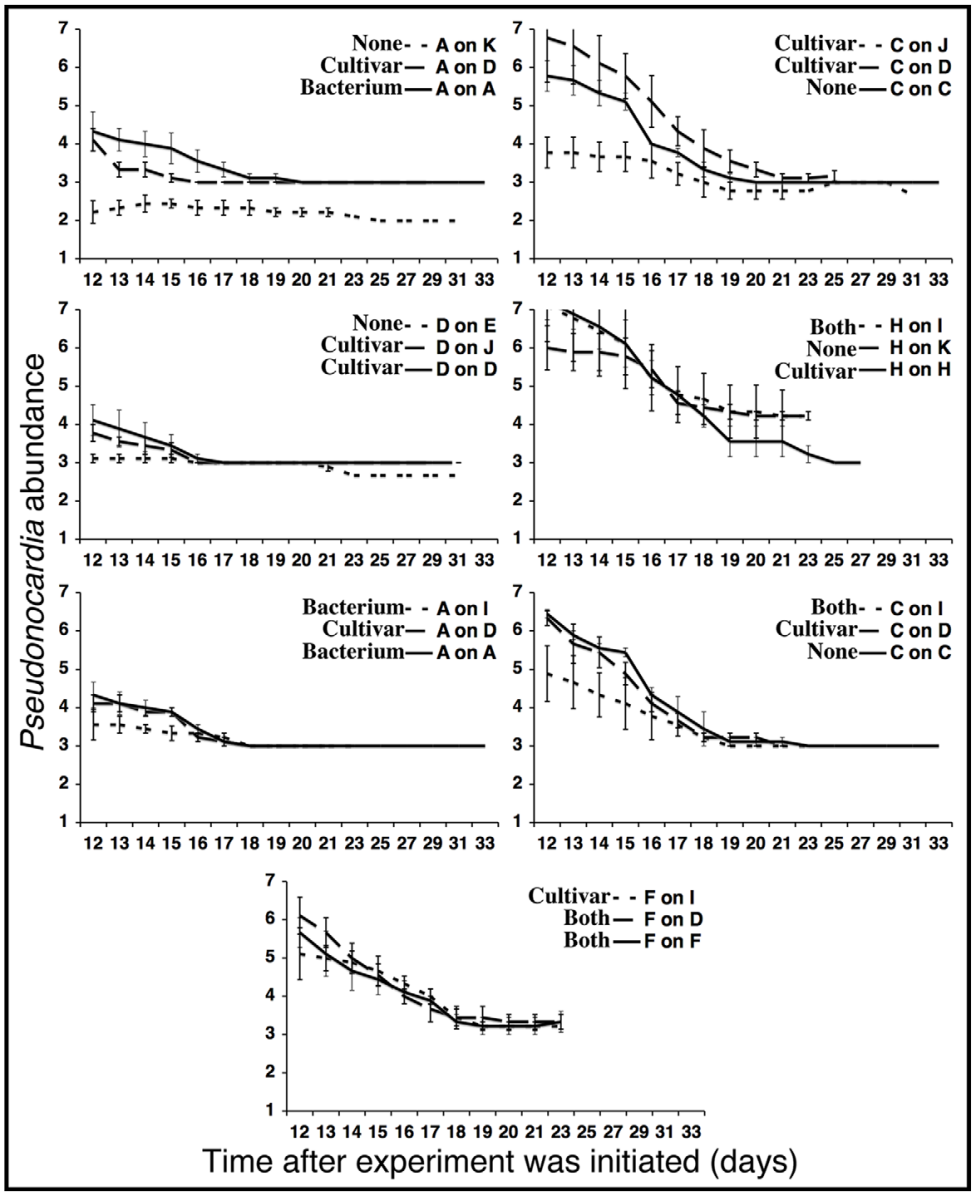
The results of the sub-colony experiment evaluating the effect of cultivar antagonisms on the abundance of *Pseudonocardia* on the cuticle of sub-colony workers. Means±SE of three sub-colonies, each containing three major workers carrying *Pseudonocardia*, are given, and values correspond to a scale from 0–12 [65]. The direction of antagonism observed *in vitro* is indicated next to the legend (see Fig. 7).

The abundance of *Pseudonocardia* on the cuticle of major workers is down-regulated during the transition from the behavioral role of garden tending early in life to foraging later [67]. Consequently, major workers in all our sub-colonies had reduced abundance of bacteria on their cuticle 11 days after the experiment was initiated (Fig. 8): the majority of ants carried low to intermediate abundances of bacteria on the cuticle (scores 3–6 in [65]). However, there was variation in coverage, with workers from one group of sub-colonies (colony A with fungus from colony K) retaining little (scores 2–3), while others retained higher (score average of 7 on the cuticle of ants from colony H with their own fungus, colony H with fungus from colony I, and colony C with fungus from colony D) visible *Pseudonocardia* cover. In all sub-colony pairings, ant cuticle coverage gradually declined from day 11 and onwards, reaching a final cover score of 2–3 by the end of the experiment (Day 33) (Fig. 8). Consequently, time, also highly significant in the overall analysis (F_15_ = 60.22, p<0.0001; Table 3), strongly affected the abundance of *Pseudonocardia* in each of the seven individual experiments (F values ranging from 6.035 to 30.01, all p<0.0001; Table 4). The ANOVAs on individual combination experiments further indicated that ant-fungus pairings had a strong effect in five of the seven experiments (Table 4), and the overall analysis confirmed significant effects of ant (F_4_ = 159.9, p<0.0001) and fungus (F_8_ = 19.85, p<0.0001) origin (Table 3). The interaction effect between time and ant-fungus pairing was only significant in one combination (ants from colony A with fungus from A, K, or D: F_20_ = 1.869, p = 0.0304; Table 4; Fig. 8). However, the interaction between ant origin and time was significant in the overall analysis (F_40_ = 3.062, p<0.0001), while fungus origin with time was not (F_80_ = 1.103, p = 0.2648, respectively; Table 3). The ANOVA showed a significant effect of *in vitro* inhibition of *Pseudonocardia* by cultivars on cuticular *Pseudonocardia* abundance (F_1_ = 10.70, p = 0.0011). However, the abundance of bacteria on the ant cuticle was larger, and not different from controls, when antagonism from the cultivar was present, while reduction in the abundance of *Pseudonocardia* (ants from colony A on fungus from colony K, ants from colony C on fungus from colony J, and ants from colony C on fungus from colony I) was only observed in combinations where there was no antagonism from the cultivar towards the actinobacterium (Fig. 8; Table 3).

## Discussion

### Symbiont-Symbiont Antagonism

Our findings show that the two mutualistic microbes of fungus-growing ants frequently but not always display antagonistic interactions, in the form of growth suppression, towards each other *in vitro*. Antagonism was observed in pairings that combined symbionts that: i) are not found together in nature (i.e., occur associated with distantly related ant hosts), ii) can associate with the same ant host, but did not originate from the same ant colony, and iii) were isolated from the same ant colony. Overall, the frequencies of pairings exhibiting inhibition were similar, with inhibition of the cultivar occurring in 70.7% and inhibition of *Pseudonocardia* occurring in 78.9% of pairings (the latter averaged across the cross-phylogeny and the within-*Acromyrmex* bioassay, where the fungus had a one-week head start). However, in general, bacterial inhibition of the cultivar was much stronger than *vice versa* (Figs. 3–6). This is perhaps not surprising because the cultivar grows slowly on artificial media, and because *Pseudonocardia*, both free-living and ant-associated ones, are well-known for their ability to produce diffusible secondary metabolites with antifungal properties [38]–[39], [68]–[69]. In addition to the formation of zones of inhibition, antagonistic discoloration was also present in some pairings, indicative of chemically mediated antagonism (cf. [62]–[63]). Consequently, only very few pairings showed no antagonistic interactions: a ZOI or discoloration was not observed in only 27.4% and 11.8% of the cultivar-*Pseudonocardia* pairings across the two bioassays, and across the cross-phylogeny and the second within-*Acromyrmex Pseudonocardia*-cultivar bioassays, respectively. Interestingly, in our cross-phylogeny evaluation of cultivar antagonism towards *Pseudonocardia*, pairings involving *Apterostigma* appeared to be more antagonistic than across all pairings, and this was asymmetric (Fig. 3). Similarly, although less clearly, cultivars associated with Argentinean *Acromyrmex* species appear to antagonize *Pseudonocardia* from Panamanian *Acromyrmex* species, and this pattern is also asymmetric (Fig. 4). Although the explanation for these patterns of antagonism remains unclear, the presence of non-random patterns, potentially dependent on the ant host (genus: Fig. 3; species: Fig. 4) or geographic location (Fig. 4), suggests underlying biologically relevant interactions that the extent of our evaluations could, however, not elucidate on.

We performed two different *Pseudonocardia-*cultivar within-*Acromyrmex* bioassay experiments, with and without a one-week head start for the cultivar, because of the presence of very strong inhibition of the cultivar by *Pseudonocardia* in the majority of the cross-phylogeny bioassay pairings. The patterns of inhibition between the two bioassays yielded complimentary results: relatively strong inhibition in a given pairing in the first bioassay typically resulted in relatively strong inhibition in the same pairing in the second bioassay (Fig. 6). However, the observed decrease in frequency of complete inhibition of cultivar growth in the second bioassay experiment (Fig. 6B) likely reflects a significant dose-response of the actinobacterial compounds on the cultivar strains. Allowing cultivars a one-week head start in the second assay reduced the time *Pseudonocardia* had to produce antibiotics, resulting in less strong inhibition (Fig. 6B). This further suggests that while the strength of inhibition observed on plates *in vitro* reflects, and may inform on the presence of antagonistic microbial interactions, caution should be taken when drawing conclusions based on the exact extent of antagonism *in vivo*.

### Impact of Cultivar-*Pseudonocardia* Antagonism on Sub-Colony Success

Our *in vitro* findings of growth suppression occurring between different combinations of cultivar and *Pseudonocardia* suggest that between-mutualist antagonism could impact the success of ant-cultivar-bacteria combinations within individual nests. In addition, the variation in the impact of antagonism observed between strains provides the opportunity to test the impact of antagonism in sub-colony experiments. In contrast to our expectations, the sub-colony experiment showed that all workers, irrespective of the extent or direction of antagonism, established their nest with the provided fungus fragment, which remained healthy for up to three weeks. Thereafter, fungus gardens in all sub-colonies started gradually declining, completely collapsing by day 31 (Fig. 7). Our statistical analysis did not support our initial hypothesis that *Pseudonocardia-*produced secondary metabolites mediate cultivar decline, as more rapid declines were not observed in sub-colonies with stronger *in vitro* antagonism of *Pseudonocardia* towards cultivars. Rather we found that time (the factor with the strongest effect), as well as ant and fungus colony origins, were important (Table 3). Other studies have noted that there is a limit to how long sub-colonies will remain stable after separation from the host colony (e.g., [37]), and our data show the same trend with control sub-colonies starting to decline at the same time as treatment sub-colonies. There are several possible explanations for these collapses, including that: i) the ants present in sub-colonies ‘give up’ on colony maintenance after a certain amount of time away from their maternal nest, ii) workers cease to take care of their fungus gardens after larvae pupate, which was likely to have occurred as a consequence of the duration of our experiment, and iii) collapses are inherently very likely to take place after sub-colonies reach a lower threshold of sustainable fungus garden mass; indeed, slow declines were observed from day 16/17 and onwards in a large proportion of pairings.

Our sub-colony findings indicate that the presence of antagonism *in vitro* from the cultivar towards *Pseudonocardia* also does not affect the success of fungus-growing ant-fungus-bacterium combinations (Fig. 7, 8). Specifically, in combinations where cultivars were paired with ants maintaining strains of *Pseudonocardia* that were inhibited by the fungus *in vitro* we found no evidence for either i) a decrease in *Pseudonocardia* abundance on the ant cuticle, or ii) significantly greater garden biomass loss. We did find a statistically significant effect of the fungus origin on *Pseudonocardia* abundance at the end of the experiment (Table 3); however, there was no evidence for a difference in the abundance of *Pseudonocardia* between control and treatment pairings (Fig. 8). Rather, a reduction in the amount of Actinobacteria on the cuticle of sub-colony workers was observed in some pairings where there was not antagonism from cultivars towards *Pseudonocardia* (Fig. 8; Table 3).

The presence of frequent antagonisms between mutualists *in vitro*, even ones originating from the same nest, without a detectable direct negative impact on the success of tripartite pairings *in vivo*, contrasts the role of antagonism in governing strain diversity of each of the individual mutualists. The potential for competition between different cultivar strains strongly impacts the ant-fungus association by precluding the maintenance of multiple fungus strains within individual nests [14]. Similarly, the rearing of only one or few *Pseudonocardia* strains within individual *Acromyrmex* nests may be selected for to avoid potential competitive conflict between different *Pseudonocardia* strains [51], [56]. We believe that there are several possible reasons for this. First, the cultivar and the *Pseudonocardia* bacteria are maintained in different locations (garden versus ant cuticle) within colonies, which could reduce direct interactions and allow for the maintenance of symbionts that would otherwise potentially display antagonism (i.e., if *Pseudonocardia* was maintained in the garden, and not on workers). This may be why fungus-growing ants maintain the two mutualists in these distinct, and somewhat isolated, locations. Second, individual strains of a mutualist (i.e., cultivar or *Pseudonocardia*) should be selected to defend their own position within nests because the introduction of another mutualist strain means that they may be replaced (or have a competitor). In contrast, within the framework of a tripartite mutualism, switching of one mutualist is not expected to compromise the fitness of the other mutualist because they do not compete for the same niche. Finally, the evolutionary history of fungus-growing ants and their mutualists appears to be shaped by frequent mutualist switches between colonies, species, and even genera [7], [48]–[53]. Having the ability to combine different strains of bacterial and fungal mutualists may benefit the ants, by allowing more flexibility for mutualist replacement after loss. Re-acquisitions after mutualist loss is known to take place in the cultivar [7], [48] and are conceivable in *Pseudonocardia*; however, this has yet to be examined.

Our findings that significant inhibition of the cultivar by *Pseudonocardia* in Petri plate bioassays did not reflect an *in vivo* negative impact on the fungus garden in sub-colony experiments contrasts with the findings of a recent study, which employed similar methods to explore *Pseudonocardia*-*Escovopsis* interactions [39]. In that study, the degree of inhibition of *Escovopsis* by *Pseudonocardia* observed *in vitro* correlated negatively with the impact of parasite infection on garden biomass loss in sub-colonies *in vivo*. However, the different findings between these two studies fits with previous work involving i) bioassays on different combinations of these symbionts [40], and ii) tests of a *Pseudonocardia*-derived compound [38]. This finding that the *Pseudonocardia*-derived antibiotics do not affect the cultivar *in vivo* supports that cultivars are unlikely to be the target of the antibiotics. Further, our findings support that *Pseudonocardia* is a mutualist of the ant-fungus association, and suggests that cultivar growth inhibition *in vitro* (this study) is due to the presence of doses of compounds exceeding what occurs in the fungus garden (see above). It is also likely that the exposure of the ants' mutualistic fungus to the small molecules of *Pseudonocardia* is likely reduced by being applied to locations of garden infection, by the worker ants rubbing *Pseudonocardia* on the site of *Escovopsis* infection [70], and perhaps by only being produced in the presence of infection (see below). Finally, it may be a worthwhile trade-off for the ants to experience some degree of cultivar inhibition if this results in significantly greater suppression of the garden parasite *Escovopsis* (i.e., garden loss from cultivar suppression is lower than garden loss due to infection). This is especially true if the ants are able to target antibiotic application to infected garden sites.

### Future Studies of the Role of Antagonism on Mutualism Stability

Our study represents a first attempt to experimentally examine whether between-mutualist interactions impact a relatively well-described insect-microbe association. A great deal of work has examined host-symbiont conflict and cooperation in the fungus-growing ant symbiosis, including: i) how ant behaviors impact mutualist diversity within nests [7], [49], [54], ii) the role of symbiont choice in shaping host-mutualist pairings [54], [71]–[72], and iii) how antagonism between genetically different mutualists may govern within-colony symbiont diversity [14], [56]. The ability to switch fungi between ant nests has greatly aided performing manipulative experiments testing questions on host-symbiont interactions in the fungus-growing ant symbiosis (e.g., [14], [49], [72]–[74]). Our findings add another layer of complexity in studying mutualist interactions in the fungus-growing ant symbiosis. However, more work is needed both in the fungus-growing ant system and in other systems to establish the role of interactions between different mutualists associating with the same host. Due to the location of *Pseudonocardia* on the ant cuticle, it is not yet possible to switch the bacterium between colonies, but if a method is developed to do so it will be possible to create, and evaluate the success of, additional ant-fungus-bacterium combination. Furthermore, our short-term (three weeks) sub-colony experiment does not rule out longer-term effects of changes due to ant-fungus-bacterium combinations, such as more subtle reductions in biomass accumulation. Finally, we evaluated the role of inhibition in healthy uninfected colonies; future studies should evaluate the role of antagonism between mutualists in the presence of *Escovopsis* and/or the number of other microorganisms known to occur in fungus gardens [41]–[43], [57]–[60]. For example, it is possible that the ants distribute *Pseudonocardia*-derived antibiotics in the fungus garden only in the presence of *Escovopsis*, which would reduce the potential impact on a susceptible cultivar in the absence of parasitism. If so, directional conflict from *Pseudonocardia* towards the cultivar could be exacerbated in the presence of the garden parasite.

## Supporting Information

Figure S1 Schematic overview of the objectives and methods of our study: the cross-phylogeny bioassay experiments (left panel), the within-*Acromyrmex* bioassay experiments (middle panel), and the sub-colony evaluation of the role of *in vitro* antagonism on stability of novel *in vivo* sub-colony ant-fungus-bacterium combinations (see main text for details).
(0.07 MB PDF)
