# Supplementary material for: Symbiont Interactions in a Tripartite Mutualism: Exploring the Presence and Impact of Antagonism between Two Fungus-Growing Ant Mutualists
Source: PLoS One. 2010 Jan 15;5(1):e8748. doi: 10.1371/journal.pone.0008748 (PMC2806923; doi:10.1371/journal.pone.0008748)
Supplement: Figure S1 — Schematic overview of the objectives and methods of our study: the cross-phylogeny bioassay experiments (left panel), the within-Acromyrmex bioassay experiments (middle panel), and the sub-colony evaluation of the role of in vitro antagonism on stability of novel in vivo sub-colony ant-fungus-bacterium combinations (see main text for details). (0.07 MB PDF) [file pone.0008748.s001.pdf]

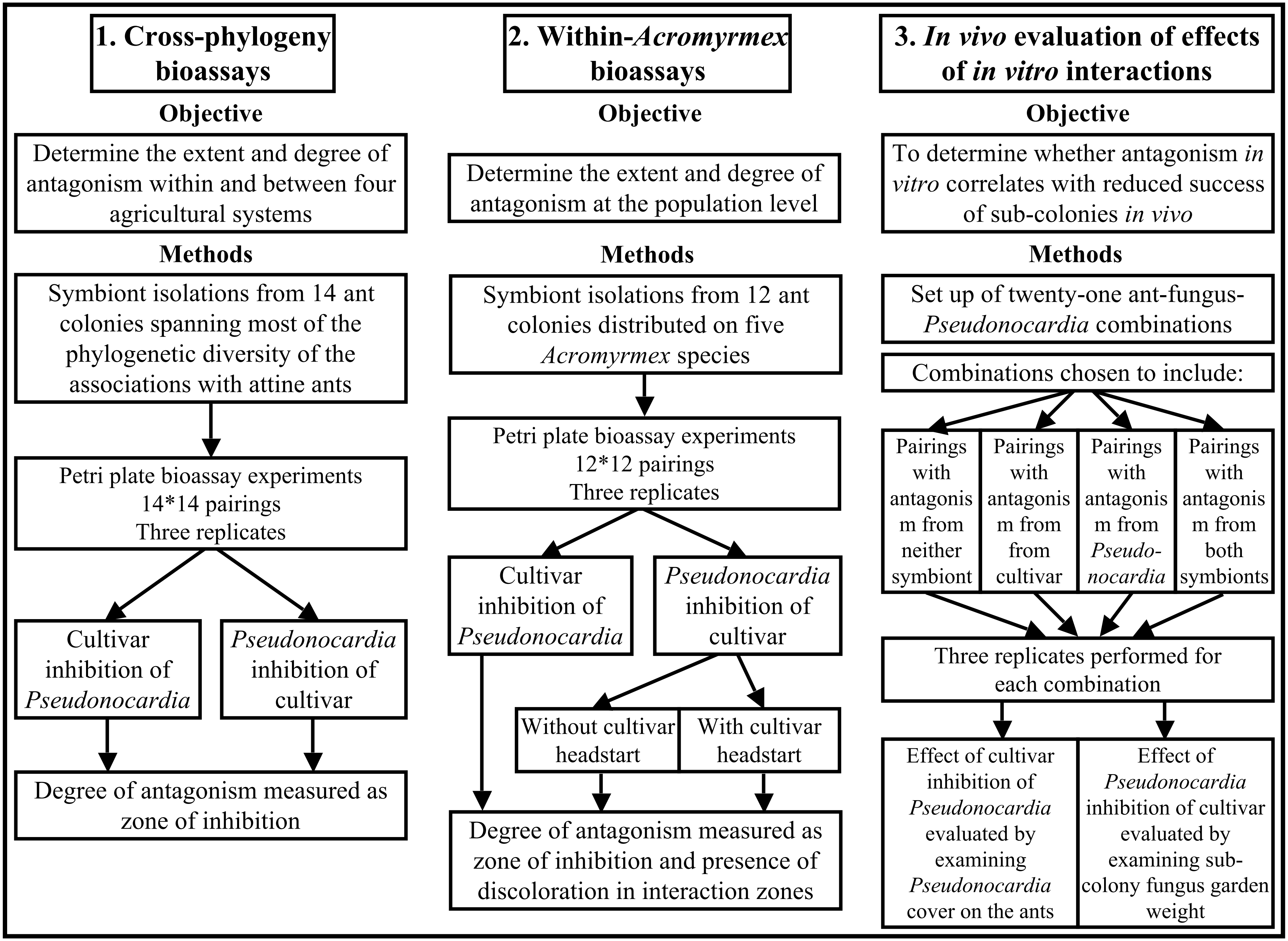
1. Cross-phylogeny bioassays
Objective
Determine the extent and degree of antagonism within and between four agricultural systems
Methods
Symbiont isolations from 14 ant colonies spanning most of the phylogenetic diversity of the associations with attine ants
Petri plate bioassay experiments 14*14 pairings Three replicates
Cultivar inhibition of *Pseudonocardia*
Pseudonocardia inhibition of cultivar
Degree of antagonism measured as zone of inhibition
2. Within-*Acromyrmex* bioassays
Objective
Determine the extent and degree of antagonism at the population level
Methods
Symbiont isolations from 12 ant colonies distributed on five *Acromyrmex* species
Petri plate bioassay experiments 12*12 pairings Three replicates
Cultivar inhibition of *Pseudonocardia*
Pseudonocardia inhibition of cultivar
Without cultivar headstart
With cultivar headstart
Degree of antagonism measured as zone of inhibition and presence of discoloration in interaction zones
3. *In vivo* evaluation of effects of *in vitro* interactions
Objective
To determine whether antagonism *in vitro* correlates with reduced success of sub-colonies *in vivo*
Methods
Set up of twenty-one ant-fungus-*Pseudonocardia* combinations
Combinations chosen to include:
Pairings with antagonism from neither symbiont
Pairings with antagonism from from cultivar
Pairings with antagonism from *Pseudo-nocardia*
Pairings with antagonism from both symbionts
Three replicates performed for each combination
Effect of cultivar inhibition of *Pseudonocardia* evaluated by examining *Pseudonocardia* cover on the ants
Effect of *Pseudonocardia* inhibition of cultivar evaluated by examining sub-colony fungus garden weight
